# 3D convolutional neural networks predict cellular metabolic pathway use from fluorescence lifetime decay data

**DOI:** 10.1063/5.0188476

**Published:** 2024-02-27

**Authors:** Linghao Hu, Daniela De Hoyos, Yuanjiu Lei, A. Phillip West, Alex J. Walsh

**Affiliations:** 1 Department of Biomedical Engineering, Texas A&M University, College Station, Texas 77843, USA; 2 Department of Microbial Pathogenesis and Immunology, School of Medicine, Texas A&M University, Bryan, Texas 77807, USA; 3 The Jackson Laboratory, 600 Main Street, Bar Harbor, Maine 04609, USA

## Abstract

Fluorescence lifetime imaging of the co-enzyme reduced nicotinamide adenine dinucleotide (NADH) offers a label-free approach for detecting cellular metabolic perturbations. However, the relationships between variations in NADH lifetime and metabolic pathway changes are complex, preventing robust interpretation of NADH lifetime data relative to metabolic phenotypes. Here, a three-dimensional convolutional neural network (3D CNN) trained at the cell level with 3D NAD(P)H lifetime decay images (two spatial dimensions and one time dimension) was developed to identify metabolic pathway usage by cancer cells. NADH fluorescence lifetime images of MCF7 breast cancer cells with three isolated metabolic pathways, glycolysis, oxidative phosphorylation, and glutaminolysis were obtained by a multiphoton fluorescence lifetime microscope and then segmented into individual cells as the input data for the classification models. The 3D CNN models achieved over 90% accuracy in identifying cancer cells reliant on glycolysis, oxidative phosphorylation, or glutaminolysis. Furthermore, the model trained with human breast cancer cell data successfully predicted the differences in metabolic phenotypes of macrophages from control and POLG-mutated mice. These results suggest that the integration of autofluorescence lifetime imaging with 3D CNNs enables intracellular spatial patterns of NADH intensity and temporal dynamics of the lifetime decay to discriminate multiple metabolic phenotypes. Furthermore, the use of 3D CNNs to identify metabolic phenotypes from NADH fluorescence lifetime decay images eliminates the need for time- and expertise-demanding exponential decay fitting procedures. In summary, metabolic-prediction CNNs will enable live-cell and *in vivo* metabolic measurements with single-cell resolution, filling a current gap in metabolic measurement technologies.

## I. INTRODUCTION

Cellular metabolism underlies cell function and behavior and, thus, is integral to normal and disease pathologies. Cancer cells often depend on glycolysis to produce energy even in the presence of oxygen, a phenomenon referred to as the Warburg effect.^1^ Furthermore, the glutaminolysis pathway can be enhanced in cancer cells to create biosynthetic precursors and compensate for reduced oxidative phosphorylation (OXPHOS) when the electron transport chain is impaired.^2^ The dependence of cancer cells on specific metabolic pathways enables cancer therapy by metabolism targeting drugs.^3,4^ Similarly, many immune cell functions are dependent on specific metabolic pathways. For example, pro-inflammatory macrophages are dependent on glycolysis, while anti-inflammatory macrophages undergo a metabolic shift toward oxidative phosphorylation.^5^ Additionally, T cells and B cells also exhibit metabolic reprogramming to be more glycolytic in activated states.^6,7^ The metabolic dependences of immune cells suggest that metabolism-modulation drugs may be effective strategies for immune therapy.^8,9^ Therefore, studies of cellular metabolism and metabolic perturbation are important for advancing fundamental and translational knowledge in many fields, including cancer biology, immunology, and therapeutics.

Tissue heterogeneity and complex cellular environments necessitate single-cell metabolic measurements.^10–12^ In particular, tumor cell metabolic heterogeneity drives different clinical responses such as therapy resistance and recurrence, hindering metabolic-based anti-cancer treatment.^13^ However, live-cell measurements of metabolism with single-cell resolution are challenging. Currently, the widely used Seahorse technology enables the detection of metabolic variations in cell populations by measuring the oxygen consumption rate (OCR) and the extracellular acidification rate (ECAR).^14^ Similarly, biochemical analyses of metabolic enzymes using Western Blot analysis, mRNA analysis, and microplate reader absorption or fluorescence assays typically require cell dissolution or fixation and cannot resolve metabolic information with single-cell resolution.^15,16^ Techniques to evaluate the metabolism of a single cell include flow cytometry, single-cell RNA sequencing, and immunofluorescence or immunohistochemistry detection of metabolic enzymes, each of which requires the destruction of cells.^17^ Therefore, noninvasive measurements of single-cell metabolism in live samples are a desirable technique and potentially beneficial for a wide range of scientific research and clinical applications.

Optical metabolic imaging provides a label-free modality to detect metabolic activities at a cellular level. This technique captures the fluorescence intensity and lifetime of autofluorescent metabolic co-enzymes, including reduced nicotinamide adenine dinucleotide (NADH). NADH is an electron acceptor in glycolysis and an electron donor in oxidative phosphorylation.^18^ Additionally, NAD^+^ is converted to NADH through reduction in glutaminolysis.^4,18^ Furthermore, NADH is used in fatty acid synthesis.^18^ NADH and its phosphate form, NADPH, have the same fluorescent excitation and emission properties, so NAD(P)H is used to represent the measured fluorescence signal of both molecules.^19^ Fluorescence lifetime imaging measures the time a fluorophore remains in the excited state before returning to the ground state by emitting a photon.^20^ The fluorescence lifetime of NADH is sensitive to the surrounding microenvironment and is altered due to conformational changes of NADH in free and enzyme-bound states. Free NAD(P)H has a short lifetime around 300–500 ps, while protein-bound NAD(P)H has a longer lifetime around 1.5–2 ns.^21^ Thus, fluorescence lifetime imaging (FLIM) can quantify changes in the free to protein-bound ratios of metabolic enzymes, and NAD(P)H FLIM metrics are often altered with metabolic perturbations in cells and tissues.^18,22^ Furthermore, fluorescence lifetime images can be segmented into individual cells, allowing for metabolic measurements at a cellular level.^23,24^

Fluorescence lifetime imaging in the time domain measures the fluorescence intensity decay as a function of time following an excitation pulse from a laser. A common FLIM technique uses time-correlated single-photon counting (TCSPC), which repeatedly records the arrival time of the emitted photons after excitation and sorts them into a histogram.^25,26^ The raw fluorescence decay data represents a temporal point spread function (TPSF) at each pixel. Traditional analysis of FLIM decay data requires deconvolution of the TPSF from a measured instrument response function (IRF) and fitting the decay to an exponential model. Due to the difference in lifetimes of free and protein-bound NAD(P)H, the fluorescence lifetime decay of NAD(P)H is often fit to a two-exponential decay model, and a mean lifetime can be calculated by the weighted average lifetime of short and long lifetimes. FLIM analysis is usually achieved using customized code or software such as SPCImage (Becker & Hickl), FluoFit (PicoQuant), FLIMfit, and FLIMJ.^26–28^ However, deconvolution and exponential fitting analysis of the decay curve requires assumptions about the data and measured signal, such as the number of lifetime components and shift in instrument response function, that necessitate domain expertise. Moreover, deconvolution and decay curve fitting are time-consuming due to the iterative nature of deconvolution and maximum likelihood estimated exponential fitting. To overcome these limitations of traditional FLIM analysis, convolutional neural networks (CNN) have been developed to generate lifetime images from raw TPSF images and intensity images at a fast speed.^29–31^

Once analyzed, the interpretation of NAD(P)H fluorescence lifetime data relative to metabolic phenotypes is difficult as a robust relationship between autofluorescence metrics and specific metabolic pathways has yet to be established. Prior studies have used conventional machine-learning algorithms to identify metabolic phenotypes of T cells and stem cells from autofluorescence lifetime features of each cell by averaging the pixel values across cellular regions.^23,32^ However, this process removes intracellular spatial patterns, which contain metabolic information since metabolic processes are distributed across mitochondria networks and the cytosol.^33^ To retain spatial fluorescence patterns, image-based convolutional neural networks (CNN) have been used to predict cell phenotypes from autofluorescence lifetime or intensity images extracted from traditional decay fitting of the TPSF.^34,35^

Despite prior advances in using machine learning to aid the interpretation of NAD(P)H fluorescence lifetime data, a 3-class prediction of metabolism phenotypes remains unexplored. Furthermore, prior models for phenotype identification use lifetime features or images extracted from traditional decay fitting and thus lose subtle spatial and temporal information that may facilitate phenotype identification. Herein, we hypothesize that a 3D (two spatial dimensions and one time dimension) CNN will identify three metabolic phenotypes of cancer cells, glycolysis, OXPHOS, and glutaminolysis. The 3D CNN will retain spatial and temporal information to increase the specificity and accuracy of metabolic pathway identification from NAD(P)H lifetime decay data. A dataset of NAD(P)H fluorescence lifetime images of MCF7 breast cancer cells with enhanced and inhibited glycolysis, OXPHOS, and glutaminolysis pathways was used to train and test the 3D CNN models. The 3D CNN models trained with NAD(P)H TPSF images discriminated three different metabolic pathways at the cellular level with more than 90% accuracy. Moreover, the 3D CNN models trained on the cancer cells were tested to predict the metabolic phenotypes of two cell lines of control and mitochondria-deficient macrophages. To our knowledge, this is the first study to successfully differentiate three major metabolic pathways, glycolysis, OXPHOS, and glutaminolysis at a cellular level using NAD(P)H autofluorescence decay data. This novel approach of NADH FLIM combined with 3D CNNs for metabolic pathway identification will enable live-cell and *in vivo* studies of metabolic heterogeneity in cancer and immunology, metabolism-targeted therapies, and genetic mitochondrial diseases.

## II. RESULTS

### A. Temporal characteristics of NAD(P)H fluorescence of cells with fixed metabolic phenotypes

**FIG. 1.**
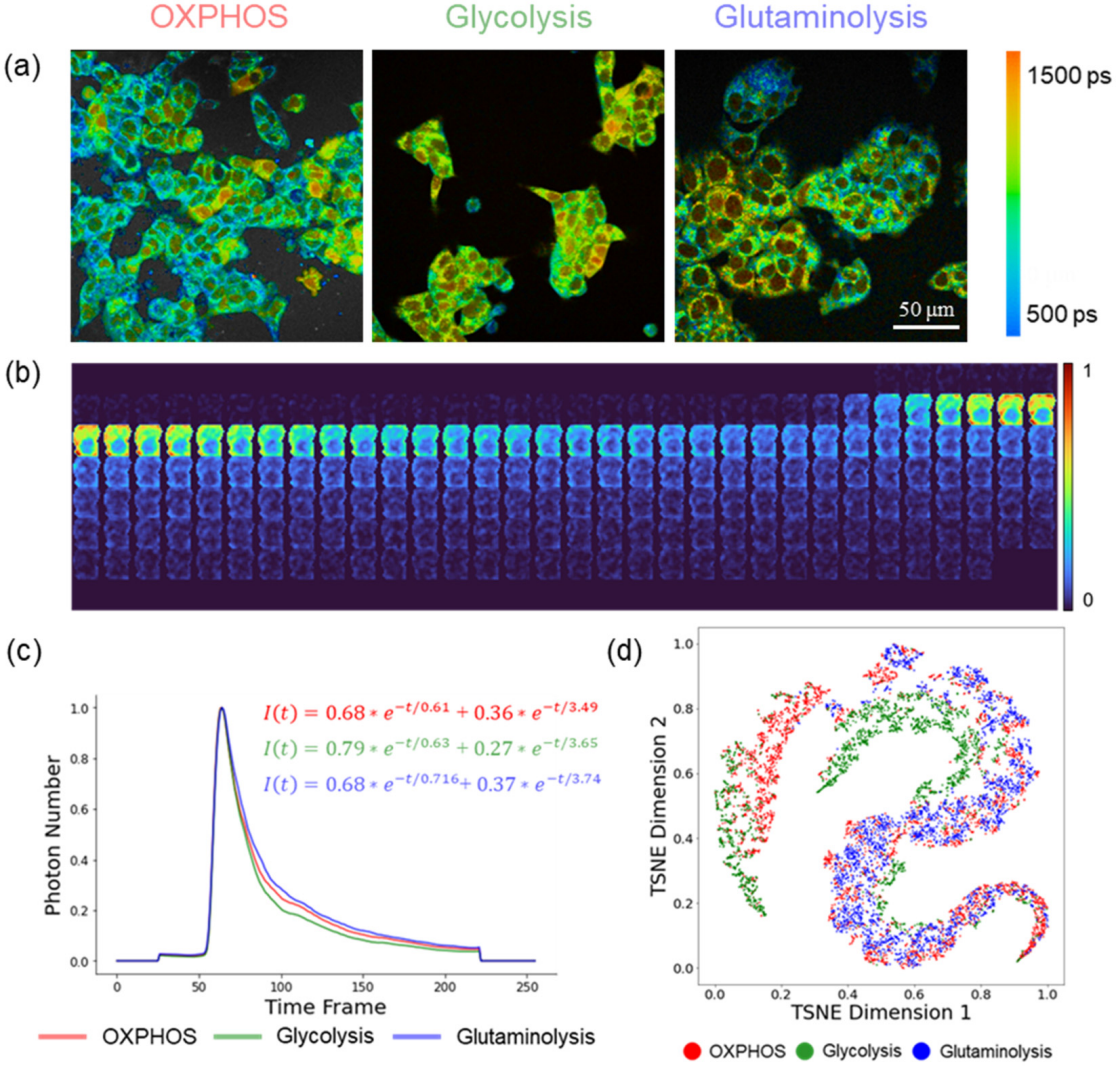
Characteristics of NAD(P)H fluorescence lifetime decays of MCF7 cells dependent on OXPHOS, glycolysis, and glutaminolysis. (a) Representative NAD(P)H *τ_m_* images of cancer cells dependent on OXPHOS, glycolysis, and glutaminolysis, scale bar = 50 *μ*m. (b) Representative fluorescent images of an MCF7 cell montaged across time. The upper left frame corresponds to t = 0 ps, and the bottom right frame corresponds to t = 12.5 ns, with 48.8 ps time resolution of each frame. The representative cell is from the glycolysis group, and the image size is 21 × 21 spatial pixels (∼22 × 22 *μ*m^2^) × 256 frames across time. (c) Average NAD(P)H decay curves (TPSF) of cells dependent on glycolysis, OXPHOS, and gluataminolysis. (The curve was obtained by averaging the decays normalized to the decay peak maximum of all pixels within a cell and then averaged across all cells within each metabolic group.) (d) t-SNE projection with the NAD(P)H intensity decay as input features of MCF7 cells dependent on glycolysis (green), OXPHOS (red), and glutaminolysis (blue).

The differences in the TPSF data across the metabolic groups can be visualized and potentially detected by machine learning models for metabolism differentiation. Representative intensity-scaled, mean fluorescence lifetime images allow visualization of different NAD(P)H fluorescence lifetimes due to metabolic perturbations at the image level [Fig. 1(a)]. The processed TPSF image size for each cell was 21 × 21 × 256 pixels (X × Y × T), and the cytoplasm contains more NAD(P)H molecules compared to the nucleus, resulting in brighter NAD(P)H intensity [Figs. 1(a) and 1(b)]. Down-sampling of the TPSF images resulted in a reduction of the NAD(P)H decay curve's length and shifted the peak position, originally mostly at the 64th or 65th (3.17 ns) [Fig. S2(b)] to the 30th or 31st time frame [Fig. S2(c) and S2(d)].

Averaged TPSF and data-dimension reduction techniques were used to visualize differences in NAD(P)H TPSF of MCF7 cells dependent on glycolytic, OXPHOS, and glutaminolysis metabolism. The average decay curves of MCF7 cells dependent on specific metabolic pathways show that the cells using glycolysis have an increased fraction of NAD(P)H with a shorter lifetime, 0.79 for glycolysis vs 0.68 for OXPHOS and glutaminolysis [Fig. 1(c)]. Furthermore, the down-sampling procedure maintained the differences in the decay curves between different metabolic groups [Figs. S2(e) and S2(f)]. To visualize the importance of the temporal information in discriminating metabolic phenotypes, the average NAD(P)H intensity within each cell at each time point was calculated resulting in 256 temporal features for each cancer cell. The t-distributed stochastic neighbor embedding (t-SNE) algorithm was applied to project these high-dimensional (256) temporal features of each cell into a 2-dimensional (2D) space to visualize the time dimension variance across the metabolic phenotypes. The t-SNE map visualizes overlap among cells using OXPHOS and glutaminolysis, and slight separation of cells using glycolysis [Fig. 1(d)].

### B. 3D CNN classifies cells using glycolysis or OXPHOS

**FIG. 2.**
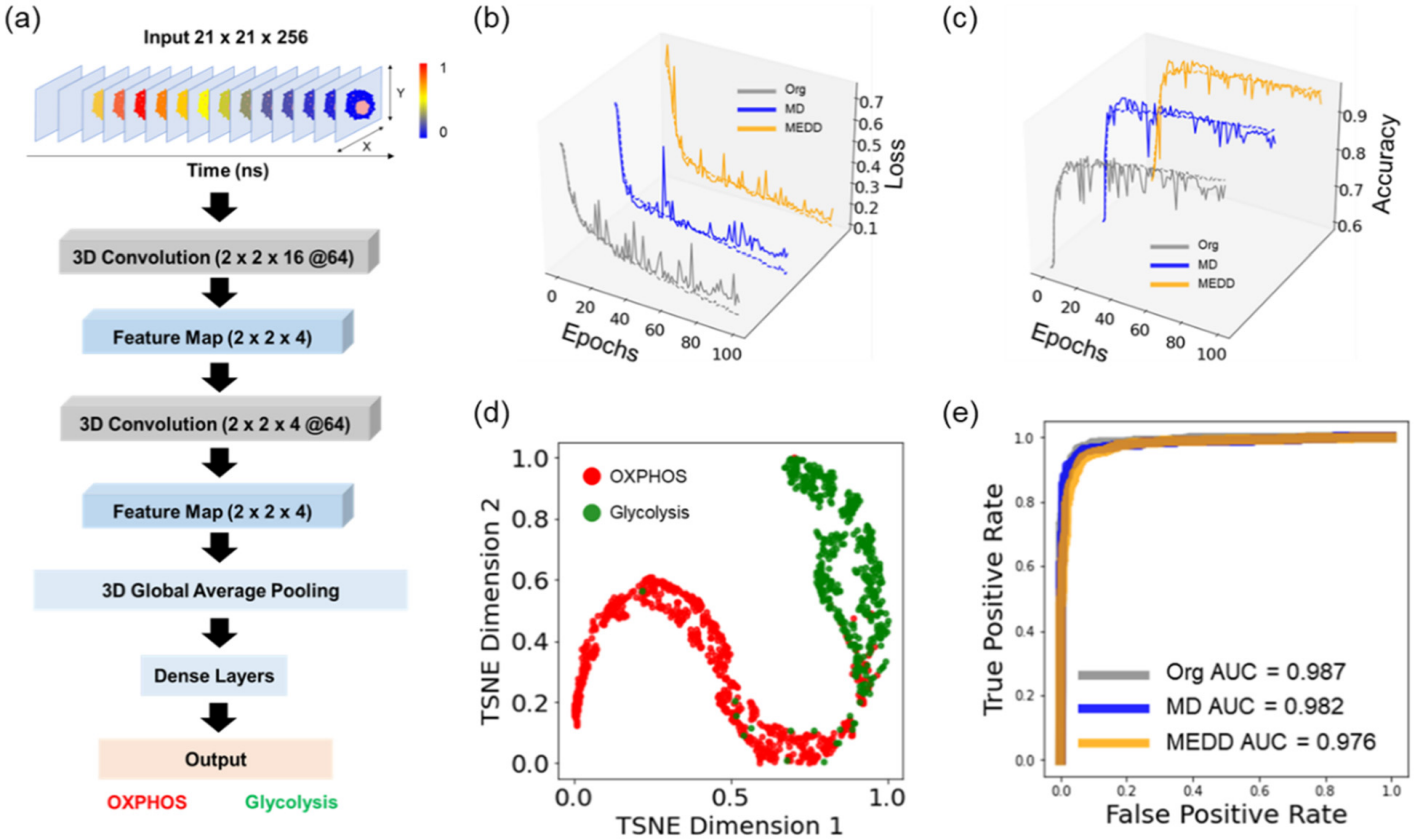
A 3D FLI-LeNet CNN model for classifying glycolytic from oxidative MCF7 cells from NAD(P)H TPSF images. (a) The structure of the FLI-LeNet CNN model for predicting cancer cells using glycolysis and cancer cells using OXPHOS based on the NAD(P)H TPSF images. (b) Validation loss and training loss by epoch for FLI-LeNet models trained with different datasets (Org: original TPSF images, MD: down-sampled TPSF images with the mean filter, MEDD: down-sampled TPSF images with the median filter). Solid lines represent validation loss, and dashed lines represent training loss. (c) Validation accuracy and training accuracy by epoch for FLI-LeNet models trained with different datasets. Solid lines represent validation accuracy, and dashed lines represent training accuracy. (d) t-SNE visualization obtained from the last activation map of the FLI-LeNet model of the test data of the model trained with the original NAD(P)H TPSF images. Each dot corresponds to one cell based on its representation in the last activation layer of the pre-trained FLI-LeNet after fine-tuning. Red data points represent cells using OXPHOS, and green data points represent the cells using glycolysis. (e) Representative ROC curves of FLI-LeNet models trained with original NAD(P)H TPSF data (Org) and down-sampled data (MD, MEDD) for predicting glycolysis or OXPHOS of MCF7 cells within the test datasets.

A 3D fluorescence lifetime imaging LeNet (FLI-LeNet) model was created to predict cancer cells as either glycolytic or oxidative [Fig. 2(a)]. The structure of the 3D FLI-LeNet model was derived from the traditional 2D LeNet model and consisted of two convolutional layers and two feature mapping layers,^36^ and the extracted feature maps allow visualization of the features used for classification. The feature map for a representative cell from the 3D FLI-LeNet model shows that both time-domain features [Figs. S3(a) and S3(b)] and morphological features including the cell edges and cytoplasm [Fig. S3(c)] provide information from the NAD(P)H TPSF images to discriminate glycolytic from OXPHOS-dependent MCF7 cells.

The 3D FLI-LeNet model was trained to differentiate glycolytic cancer cells from oxidative cancer cells using the NAD(P)H TPSF images (21 × 21 × 256) with a 0.001 learning rate. After approximately 30 epochs, the model attained a validation loss below 0.1 [Fig. 2(b)] and a validation accuracy of around 90% [Fig. 2(c)]. Moreover, the models trained with MD and MEDD showed a similar training process [Figs. 2(b) and 2(c)]. To further visualize the training performance, 64 learned features extracted from the FLI-LeNet model were projected onto a 2D space using t-SNE. The FLI-LeNet identified features that separated the cells using glycolysis from those using OXPHOS, as evidenced by their well-separated clusters in the t-SNE plot [Fig. 2(d) and Figs. S4(b) and S4(c)]. As a result, the FLI-LeNet model trained with the original TPSF images achieved an average AUC ROC of 0.978, an average accuracy of 92.0%, an average recall of 88.3%, and an average precision value of 97.4% for the fivefold test datasets in classifying MCF7 cells using glycolysis vs those using OXPHOS [Fig. 2(e), Table I, Table S3].

**TABLE I.** Performance of FLI-LeNet CNN model on prediction of glycolytic and oxidative cells trained with different datasets. Values are mean +/− standard deviation for the test datasets of the fivefold cross-validation replication.

Data type	AUC	Accuracy	Precision	Recall
Org^a^	0.978 (±0.003)	92.0% (±2.2%)	97.4% (±0.5%)	88.3% (±9.2%)
MEDD^b^	0.978 (±0.006)	92.7% (±1.6%)	97.8% (±0.3%)	85.7% (±4.6%)
MD^c^	0.980 (±0.004)	91.8% (±1.2%)	97.6% (±0.5%)	83.0% (±2.9%)

^a^ Org: original TPSF dataset (21 × 21 × 256).

^b^ MEDD: down-sampled TPSF dataset (21 × 21 × 128) with median filter.

^c^ MD: down-sampled TPSF dataset (21 × 21 × 128) with mean filter.

The FLI-LeNet model trained with the temporal down-sampled TPSF images maintained the ability to distinguish metabolic status with comparable prediction performances and a training speed that is twice as fast as the FLI-LeNet model trained with the original TPSF images. The FLI-LeNet model trained with MEDD attained an AUC value of 0.978, an accuracy of 92.7%, a recall of 85.7%, and a precision of 97.8% in predicting glycolytic and oxidative MCF7 cells [Fig. 2(e), Table I, Table S4]. Similarly, the FLI-LeNet model trained with MD achieved an accuracy of 91.8%, an AUC of 0.980, a precision of 97.6%, and a recall of 83.0% [Fig. 2(e), Table I, Table S5].

### C. 3D CNN models allow differentiation of glycolysis, OXPHOS, and glutaminolysis metabolic pathways in cancer cells with NAD(P)H TPSF images

**FIG. 3.**
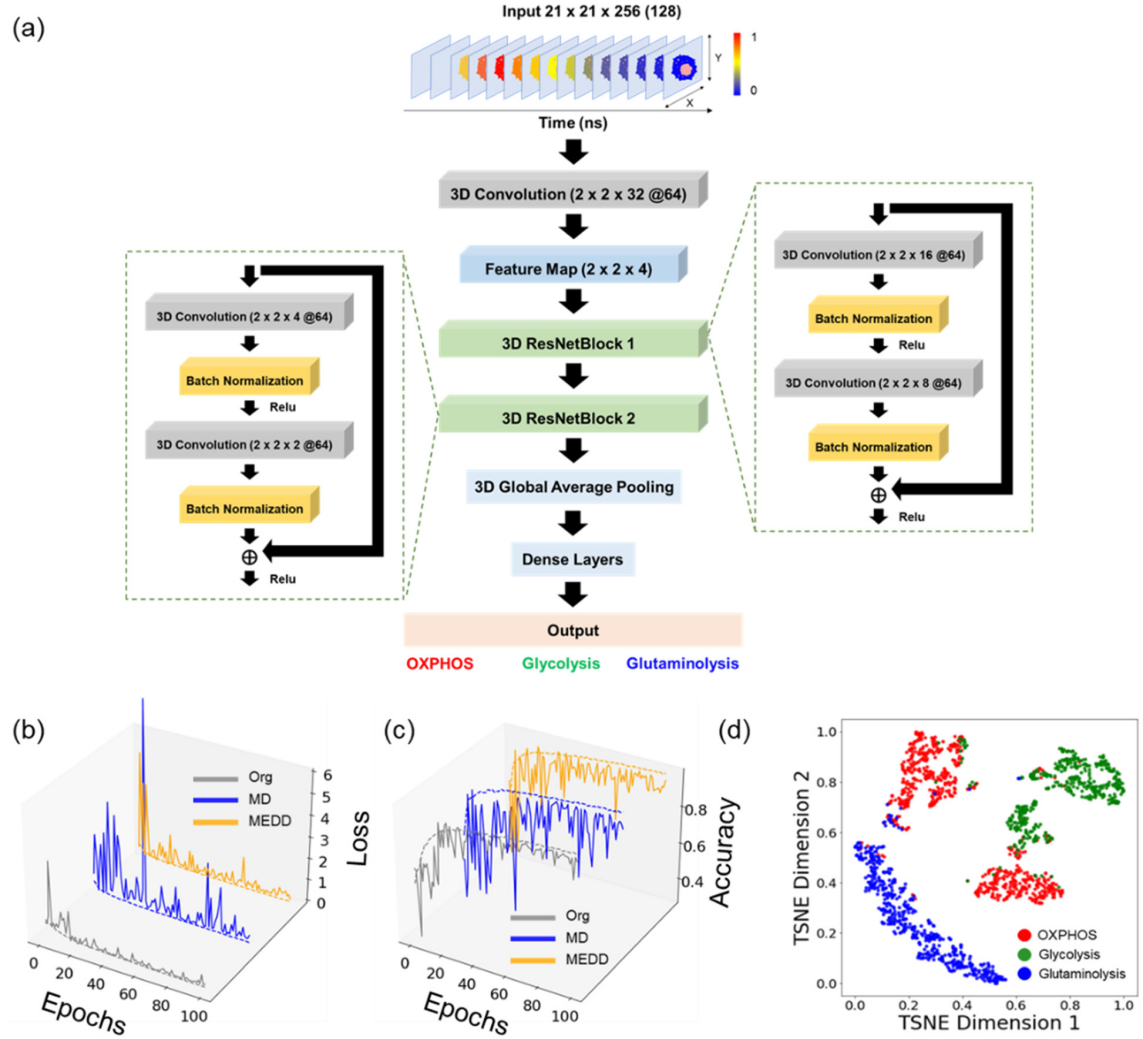
3D FLI-ResNet CNN model for classifying MCF7 cells as dependent on glycolysis, OXPHOS, and glutaminolysis. (a) Illustration of the structure of the FLI-ResNet CNN model for predicting cancer cells using glycolysis, OXPHOS, and glutaminolysis from the original NAD(P)H TPSF images. (b) Validation and training loss by epochs for the FLI-ResNet models trained with a 0.001 learning rate for predicting metabolic activity in different datasets (Org: original TPSF images, MD: down-sampled TPSF images with the mean filter, and MEDD: down-sampled TPSF images with the median filter); solid lines represent validation loss, and dashed lines represent training loss. (c) Validation accuracy and training accuracy over epochs for FLI-ResNet trained with a 0.001 learning rate for predicting metabolic activity in different datasets; solid lines represent validation accuracy, and dashed lines represent training accuracy. (d) 2D t-SNE visualization of the test data of the last activation map of the FLI-ResNet model created with the original NAD(P)H TPSF images. Red data points represent MCF7 cancer cells dependent on OXPHOS, green data points represent MCF7 cancer cells dependent on glycolysis, and blue data points represent MCF7 cancer cells dependent on glutaminolysis.

To further explore the ability of 3D CNN models to detect metabolic activities in cells from NAD(P)H fluorescence lifetime images, we expanded our dataset to include three groups of MCF7 cells, each dependent on a single metabolic pathway: glycolysis, OXPHOS, or glutaminolysis. A new 3D CNN model called FLI-ResNet was developed for the prediction of glycolysis, OXPHOS, and glutaminolysis from NAD(P)H TPSF images and compared with three-group prediction performance of FLI-LeNet models [Fig. 3(a)]. The feature map of a representative cell showed that the FLI-ResNet model extracts both temporal and spatial patterns in the TPSF images (Fig. S5).

Both the FLI-LeNet and FLI-ResNet models were trained on the original TPSF images of cells using a learning rate of 0.001. After approximately 30 epochs, the FLI-ResNet models achieved a validation loss below 0.1, with a validation accuracy exceeding 85% [Figs. 3(b) and 3(c)]. In comparison, the FLI-LeNet models attained a validation loss below 0.1 and a validation accuracy above 80% after 30 epochs [Figs. S6(c) and S6(d)]. It was observed that the FLI-ResNet exhibited a stable performance with fewer fluctuations in validation accuracy and loss during the training progress when trained with the original TPSF images than when trained with MD and MEDD [Figs. 3(b) and 3(c)]. To further assess the ability of the models to identify cell dependencies on glycolysis, OXPHOS, and glutaminolysis, t-SNE dimensionality reduction algorithms were applied to the last activation layer, enabling the visualization of cell clustering in a 2D space based on the extracted features of the models. The t-SNE maps of the last activation layer of the FLI-ResNet and FLI-LeNet models show the separation of MCF7 cancer cells dependent on glycolysis, OXPHOS, and glutaminolysis and subgroups of cells within the glycolysis and OXPHOS dependent populations [Fig. 3(d), Figs. S6(e) and S7].

Both the FLI-ResNet and FLI-LeNet models discriminate MCF7 cells with differing metabolic activities with an accuracy above 85%. The FLI-ResNet model trained on the original TPSF images showed the best performance in differentiating MCF7 cells using glycolysis, OXPHOS, or glutaminolysis, with an average accuracy of 92.6%, precision of 92.6%, recall of 93.1%, and an F1-score of 92.7% for the fivefold cross-validation (Table II, Table S6). In contrast, the FLI-LeNet model trained on the original TPSF images achieved an average accuracy, precision, recall, and F1-score of 85.0%, 86.7%, 85.4%, and 85.3%, respectively (Table II, Table S9). When trained on down-sampled datasets, the FLI-ResNet model achieved recall, precision, and F1-scores of 87%–89% for distinguishing metabolic activities of MCF7 cells from NAD(P)H TPSF images (Table II, Tables S7 and S8). The FLI-LeNet models trained on the MD and MEDD down-sampled datasets maintained similar performance, achieving accuracy rates of ∼88% and precision, recall, and F1-scores of 88%–89% (Table II, Tables S10 and S11).

**TABLE II.** Performance of FLI-ResNet and FLI-LeNet CNN models on prediction of cells using glycolysis, OXPHOS, and glutaminolysis trained with different datasets. Values are mean +/− standard deviation for the test datasets of the fivefold cross-validation replication.

Data type	Model	Accuracy	Precision	Recall	F1-score
Org^a^	FLI-ResNet	92.6% (±2.1%)	92.6% (±2.2%)	93.1% (±1.8%)	92.7% (±2.1%)
MEDD^b^	FLI-ResNet	87.0% (±4.9%)	89.4% (±4.1%)	87.5% (±4.0%)	87.4% (±4.7%)
MD^c^	FLI-ResNet	88.0% (±5.0%)	89.1% (±3.6%)	89.5% (±4.1%)	88.0% (±5.1%)
Org^a^	FLI-LeNet	85.0% (±3.6%)	86.7% (±2.1%)	85.4% (±4.2%)	85.3% (±3.6%)
MEDD^b^	FLI-LeNet	88.1% (±1.8%)	89.0% (±1.3%)	88.7% (±1.9%)	88.4% (±1.7%)
MD^c^	FLI-LeNet	88.6% (±1.2%)	89.3% (±1.6%)	88.8% (±0.7%)	88.9% (±1.2%)

^a^ Org: original TPSF dataset (21 × 21 × 256).

^b^ MEDD: down-sampled TPSF dataset (21 × 21 × 128) with median filter.

^c^ MD: down-sampled TPSF dataset (21 × 21 × 128) with mean filter.

### D. The metabolic-prediction models transfer to a FLIM dataset of murine macrophages with genetically modulated mitochondria function

**FIG. 4.**
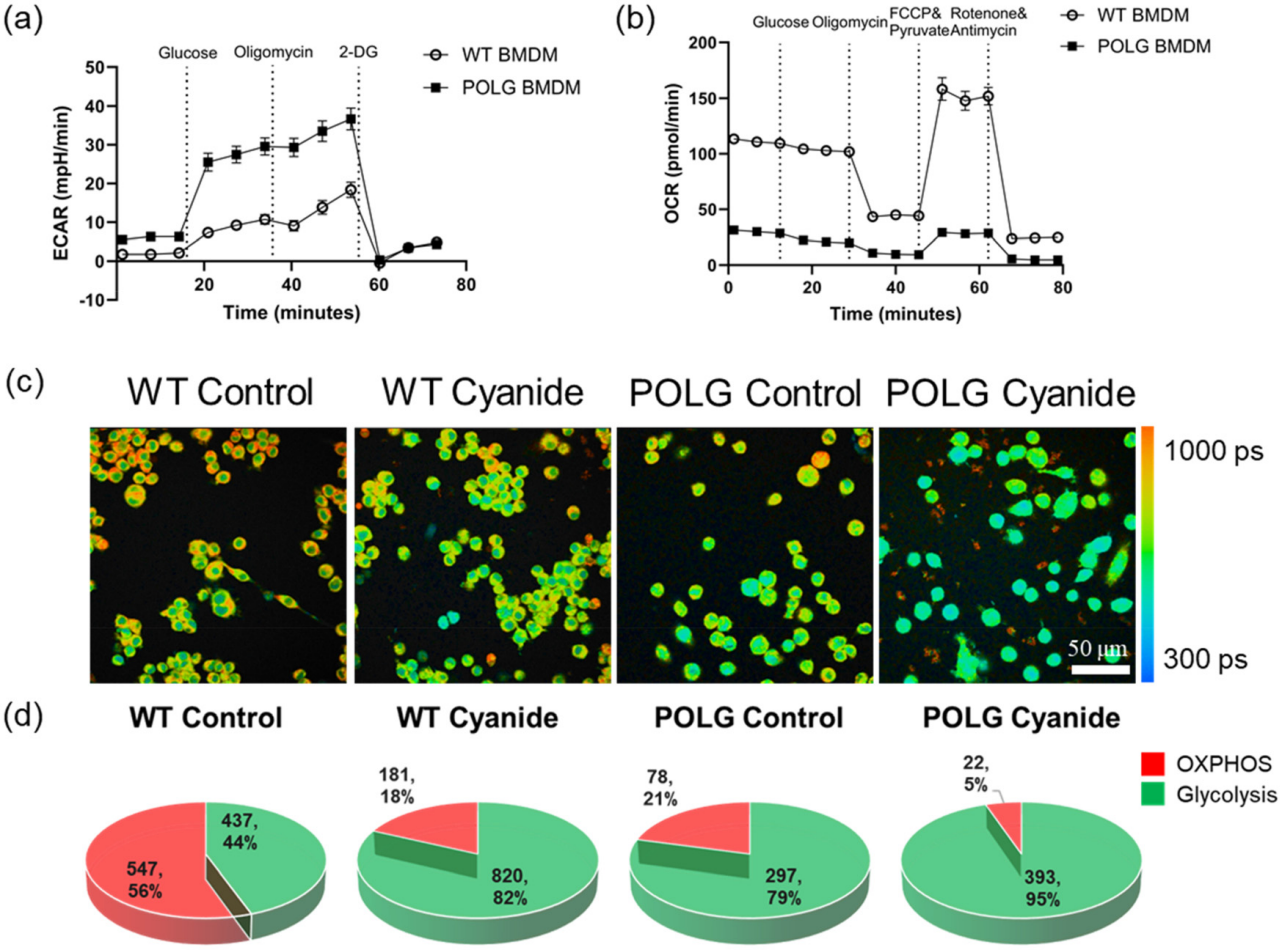
Prediction of metabolic activity in WT and POLG-mutated murine macrophages using 3D FLI-LeNet CNN models. (a) Extracellular acidification rate (ECAR) of WT BMDMs and POLG-mutated BMDMs was measured under basal conditions and with the sequential addition of glucose, oligomycin, and 2-DG. (b) Oxygen consumption rate (OCR) of WT BMDM and POLG-mutated BMDMs was under basal conditions and with the sequential addition of glucose, oligomycin, FCCP & pyruvate, and rotenone & antimycin. (c) Representative NAD(P)H *τ_m_* images of control and cyanide-treated WT and POLG macrophages show alterations in mean fluorescence lifetimes due to POLG mutation and cyanide treatment, scale bar = 50 *μ*m. (d) Prediction of the metabolism of WT and POLG BMDMs as glycolysis or OXPHOS by the FLI-LeNet models trained with MCF7 cancer cells using the original TPSF images. Data are the number of cells and corresponding percentage.

The applicability of the 3D CNN models was evaluated using wild-type (WT) and POLG-mutated murine BMDMs, which have mitochondrial DNA mutations that result in mitochondria and OXPHOS dysfunction.^37^ The sequential addition of glucose followed by the electron transport chain inhibitor oligomycin stimulated the maximal glycolytic rate resulted in an increase in ECAR in both WT and POLG BMDMs [Fig. 4(a)]. The addition of 2-DG to inhibit glycolysis decreased ECAR of both WT and POLG BMDMs [Fig. 4(a)]. In subsequent experiments of measuring OCR, the successive addition of FCCP plus pyruvate and rotenone plus antimycin to stimulate and inhibit OXPHOS resulted in an increase and decrease, respectively, of the OCR of WT BMDMs [Fig. 4(b)]. However, the changes in OCR of POLG BMDMs exposed to FCCP plus pyruvate and rotenone plus antimycin were not as great in magnitude as compared with the WT BMDMs, indicating impairments in OXPHOS capacity of POLG BMDMs [Fig. 4(b)]. In the presence of glucose, oligomycin caused a decrease in OCR in WT BMDM [Fig. 4(b)]. However, this decline was less pronounced in POLG BMDMs. Moreover, the POLG BMDMs exhibited elevated basal levels of ECAR and reduced levels of OCR compared to the WT BMDMs, suggesting that the POLG BMDMs are more glycolytic than the WT BMDMs as we previously reported [Figs. 4(a) and 4(b)].^38^

NAD(P)H fluorescence lifetime imaging revealed different NAD(P)H lifetime characteristics of the WT and POLG BMDM [Fig. 4(c)]. WT BMDMs exhibited a longer NAD(P)H lifetime compared to the POLG-mutated macrophages [Fig. 4(c), Fig. S8]. Treatment with cyanide decreased the mean NAD(P)H lifetime of both WT and POLG BMDMs [Fig. 4(c), Fig. S8].

NAD(P)H FLIM images of control and cyanide-treated WT and POLG BMDMs were input into the glycolysis vs OXPHOS FLI-LeNet models previously trained with MCF7 cancer cell images. For the control WT BMDMs, the FLI-LeNet model (original data, 256 images in the time dimension) predicted 56% as oxidative and 44% as glycolytic [Fig. 4(d)]. After treatment with cyanide to inhibit OXPHOS and stimulate glycolysis, 82% of the WT macrophages were predicted to be glycolytic, and 18% were oxidative [Fig. 4(d)]. In contrast, 79% of control POLG BMDMs were predicted to be glycolytic and 21% oxidative [Fig. 4(d)] at rest. After treatment with cyanide, 95% of the POLG macrophages were identified as glycolytic [Fig. 4(d)] and 5% as oxidative. The FLI-LeNet models generated from MEDD and MD data resulted in similar predictions of the WT macrophages, with an increase in the glycolytic portion observed with cyanide treatment (Fig. S9). The MD FLI-LeNet model identified that most POLG BMDMs were glycolytic, and cyanide treatment of POLG BMDMs resulted in a smaller proportion of glycolytic macrophages (Fig. S9). However, the FLI-LeNet model trained with MEDD data predicted a majority of POLG BMDMs to be oxidative for both the control and cyanide-treated groups (Fig. S9).

## III. DISCUSSION

Optical imaging of the fluorescent co-enzyme NAD(P)H is a functional, label-free technique to assess metabolic perturbations.^20,21,24,39–44^ However, the interpretation of variations in the autofluorescence lifetime metrics is challenging and requires domain expertise since NAD(P)H lifetime measurements are multivariant, and many distinct metabolic pathways contribute to NAD(P)H signals. Recent studies have employed machine-learning algorithms to identify cellular phenotypes from autofluorescence intensity and lifetime features;^23,32,33,45^ however, the separation of three metabolic states, glycolysis, OXPHOS, and glutaminolysis, from autofluorescence lifetime data is difficult due to the intricate interconnections of metabolic pathways, and the limited information contained within lifetime features extracted by exponential fitting. This study explores deep learning models for a three-way prediction of cancer cell metabolism as dependent on glycolysis, OXPHOS, or glutaminolysis from NAD(P)H fluorescence lifetime decay data.

The novel 3D models presented here, FLI-LeNet and FLI-ResNet, simplify fluorescence lifetime image analysis and have the potential to be used for metabolic profiling of live cells from NAD(P)H FLIM images. Previously, pre-trained CNN models have been used to generate lifetime component images directly from the lifetime data without deconvolution and fitting; however, these models may be limited to the lifetime values and characteristics of the training dataset and report that lifetime values are not metabolic functions of cells.^29,30^ Herein, the 3D FLI-LeNet and FLI-ResNet CNN models directly output metabolic activities of cells using the raw TPSF data and bypassing traditional FLIM analysis techniques. Compared to 2D CNNs, 3D CNNs allow an additional dimension of input data, allowing the models to capture both temporal and spatial dynamics within the X-Y-T NAD(P)H TPSF images. The t-SNE visualization of the temporal features of cells showed a slight separation of glycolytic cells from cells using OXPHOS and glutaminolysis, implicating that temporal patterns within the fluorescence decay can effectively discriminate different metabolic activities [Figs. 1(c) and 1(d)]. Furthermore, spatial signals within NAD(P)H lifetime images encode metabolic information, and previous analysis of mitochondria structure in NAD(P)H images revealed different cluster patterns in glycolytic and oxidative cells.^33,46,47^ The FLI-LeNet enabled the classification of the glycolytic and oxidative phenotypes of cancer cells with over 90% accuracy, which was comparable to 2D CNN models that use five NAD(P)H lifetime component images (*τ_1_*, *τ_2_*, *α_1_*, *τ_m_*, intensity).^35^ The recall of the FLI-LeNet was lower than the precision and accuracy values for predicting glycolysis and OXPHOS utilization by breast cancer cells, possibly indicating heterogeneity in the response of MCF7 cancer cells to glycolysis inhibition by 2-DG.^48^ The best-performing model was the FLI-ResNet trained with the original NAD(P)H TPSF images, achieving 94% accuracy in differentiating cells using glycolysis, OXPHOS, and glutaminolysis (Table II).

Although more powerful than 2D CNN models, 3D CNNs are computationally more expensive, resulting in longer training and inference times. Herein, the 3D TPSF NAD(P)H images are composed of 256 2D intensity images representative of different time points, demanding 30 times the storage memory (∼400 MB vs 12.7 MB) and 1.2 times the computational cost (144 690 vs 119 466 parameters) for around 5000 images, as compared to the previous 2-D CNN model trained with NAD(P)H lifetime images. To overcome this challenge, protocols were developed to down-sample the TPSF images in the time dimension by applying mean and median filters with a window of 3 to halve the original dataset (Fig. S2). The FLI-LeNet models trained with the down-sampled datasets performed similarly to models trained on the original TPSF images but trained three times faster. However, the FLI-LeNet model trained with MEDD did not perform well when applied to the macrophage datasets, suggesting the quality of training data is sensitive to median down-sampling (Fig. S9). The differences in MD and MEDD FLI-LeNet performance (Fig. S9) may be attributed to differences in variable effects of mean vs median averaging on the noise within the lifetime decay curves. The FLI-ResNet trained with original TPSF images exhibited a better performance compared to models trained with MD and MEDD. The FLI-ResNet model, with its deeper network and residual connections, may capture more intricate and abstract features than the FLI-LeNet model when sufficient training resources are available^49^ and, thus, be more sensitive to information lost with down-sampling.

To ensure the versatility of the metabolism-prediction models beyond MCF7 cells and broaden their applicability to various cell types and studies, we applied the FLI-LeNet model to NAD(P)H FLIM data of murine macrophages with and without the POLG mutation. The polymerase gamma (POLG) is the enzyme responsible for replicating and maintaining mitochondrial DNA (mtDNA) within the mitochondria.^37^ Mutations in the POLG gene are associated with a range of disorders characterized by mtDNA instability, leading to reduced fidelity and efficiency of mtDNA replication.^50,51^ These mutations result in mitochondrial dysfunction, which affects cellular metabolism. Therefore, WT and POLG BMDMs provide a model with known metabolic phenotypes to assess the efficacy of the 3D CNN models. The pre-trained FLI-LeNet correctly predicted a higher fraction of POLG BMDMs as glycolytic than the WT macrophages [Fig. 4(d), Fig. S9, Org and MD models], a finding that is consistent with ECAR and OCR data of the differences in the basal metabolic states of WT and POLG BMDMs [Figs. 4(a) and 4(b), Org and MD models]. Additionally, the FLI-LeNet identified an increased fraction of glycolytic WT BMDMs with cyanide treatment, consistent with the expected metabolic shift due to OXPHOS inhibition [Fig. 4(d), Fig. S9, Org and MD models]. Even though an elevated fraction of glycolytic phenotypes in POLG BMDMs upon cyanide treatment was also predicted by FLI-LeNet [Fig. 4(d), Fig. S9, Org and MD models], this increase is less compared to the WT BMDMs [Fig. 4(d), Fig. S9, Org and MD models]. This observation indicates that the effectiveness of cyanide in compromising OXPHOS in macrophages with POLG mutation is reduced when compared to WT macrophages.

The applicability of FLI-LeNet models to NAD(P)H fluorescence lifetime images of BMDMs suggests that the features identified by the models trained with cancer cells are preserved across datasets of different species and different metabolic perturbations. The model is primarily influenced by the temporal patterns in NAD(P)H fluorescence lifetime images, and the similarity in morphological characteristics, such as cell size and intensity difference between the cytoplasm and nucleus [Figs. 1(a) and 4(c)], among BMDMs and MCF7 cells likely also facilitates the application of cancer cell-trained FLI-LeNet CNN models to macrophages. The accurate results of the FLI-LeNet model on the BMDMs indicate the successful transferability of the FLI-LeNet model developed with data of MCF7 cells with glycolysis and OXPHOS inhibitor treatment and glucose, and pyruvate substrate exposure to genetic modulation-induced metabolic data.

In this study, the lifetime decay matrix was obtained using time-correlated single-photon counting (TCSPC) with a time resolution of 12.5 ns and 256 time frames. The lifetime decay matrix can also be measured using time-gated imaging or pulse sampling with fewer time frames.^52^ It is possible that a 3D CNN trained with different formats of decay data can effectively distinguish metabolic phenotypes of cells, as the down-sampled NAD(P)H TPSF images yielded similar performance for identifying metabolic pathways (Figs. 2 and 3). Although the ResNet 3D CNN achieved high accuracy in separating MCF7 cells dependent on glycolysis, OXPHOS, and glutaminolysis, metabolic pathways are not mutually exclusive. Significant crosstalk and overlap between different metabolic pathways can exist within a cell. In this study, the training datasets contained cells with carefully controlled metabolic activities altered by nutrients within the media and inhibition of pathways by chemicals. The NAD(P)H TPSF images likely contain features that could be informative on the heterogeneity of metabolic pathway use by cells, and CNNs have the potential to handle more challenging tasks, such as mapping the percentage of energy supplied by different metabolic pathways. However, the advancement of CNN models for this application to separate contributions of metabolic activities within individual cells is primarily hindered by the limited availability of ground truth single-cell data. Obtaining high-quality and comprehensive datasets that accurately represent various metabolic conditions at the cellular level would greatly facilitate the further development and refinement of CNN models for metabolism analysis.

The classification of cellular metabolic activities from NAD(P)H fluorescence decay image using 3D CNNs may be useful for diverse applications that require non-contact, live-cell detection of metabolic states at the cellular level. The models presented here were developed and validated using *in vitro* cancer cell experiments, with cellular metabolism manipulated chemically. Although the models demonstrated accurate transfer to NAD(P)H FLIM images of macrophages with genetic metabolic perturbations, there are likely limitations of the models for application to more complex samples such as tissues imaged *ex vivo* or *in vivo*. For clinical cancer applications, the tumor environment is complex, including cancer cells with heterogeneous metabolic activities and drug responses and additional cell populations such as immune cells, fibroblasts, and endothelial cells. While the models described herein may lack direct applicability to images of such clinical tissues, the models could be retrained using representative *in vivo* and *ex vivo* tissue data. As the number of prediction outcomes increases to encompass additional cell types and metabolic states, the volume of data required for training also increases. To overcome the challenges of obtaining sufficient *in vivo* and *ex vivo* FLIM data, training datasets could include simulated FLIM images where lifetime parameters, cell and nucleus size, morphology, and intercellular and intracellular heterogeneity are selected to mimic experimental data.^53^ Future studies may expand the applicability of FLIM prediction models by training with various biological samples to create an ensemble of models specific to the characteristics of each FLIM dataset.

## IV. CONCLUSION

In this paper, the predominance of three major metabolic pathways, glycolysis, oxidative phosphorylation, and glutaminolysis, in MCF7 cancer cells was predicted from NAD(P)H TPSF images using 3D CNN models. The FLIM-based CNN models effectively utilize both temporal and spatial information within the NAD(P)H TPSF data, achieving accuracy rates exceeding 90%. Notably, the CNN models trained with human cancer cells were successfully transferred to murine macrophages. In conclusion, the combination of autofluorescence lifetime imaging of NAD(P)H and 3D CNN models offers a label-free modality for identifying and characterizing metabolic activities in live cells that can be promoted across different metabolic perturbations and various cellular contexts for broad applications.

## V. METHODS

### A. NAD(P)H fluorescence lifetime dataset of cancer cells

The NAD(P)H fluorescence lifetime images of MCF7 cancer cells with different metabolic activities were provided by Hu and Walsh. The methods summarized here are covered in full detail in Hu *et al.*^35^ MCF7 breast cancer cells were cultured in the Dulbecco's Modified Eagle's Medium (DMEM) with glucose (50 mM), pyruvate (2 mM), 1% antibiotic-antimycotic, and 10% fetal bovine serum (FBS). The cells were seeded at a density of 2 × 10^5^ cells in 2 ml of the culture media per 35 mm glass-bottom imaging dish 48 h before imaging. Three metabolic groups were created to target glycolysis, oxidative phosphorylation (OXPHOS), and glutaminolysis. To enhance glycolysis, the cells were treated with NaCN (4 mM) to inhibit OXPHOS 5 min before imaging. To enhance OXPHOS, 2-Dexoy-D-glucose (2-DG, 50 mM) was added to the cells 1 h before imaging to inhibit glycolysis. Additionally, a second group of OXPHOS enhanced cells was created by providing glucose-starved cells with glucose-free DMEM supplemented with pyruvate (50 mM), 1 h prior to imaging. To enhance glutaminolysis, cells were plated in DMEM with glutamine (2 mM) as the only nutrient and imaged at 1, 2, and 3 h. NAD(P)H fluorescence lifetime images were captured on a multiphoton fluorescence lifetime microscope (Marianas, 3i) using 750 nm excitation and a bandpass filter (447/60 nm) to isolate emission. Fluorescence lifetime decays of each cell were obtained through cell segmentation in CellProfiler,^54^ using an automated image segmentation pipeline previously described.^55^ The number of cancer cells in each metabolic group is summarized in Table S1.

### B. NAD(P)H lifetime imaging of POLG macrophages

Experimental details for the polymerase gamma (POLG) murine bone marrow-derived macrophage (BMDM) experiments including isolation of cells, cell culture, and Seahorse metabolic flux assay are fully described in the supplementary material. For NAD(P)H lifetime imaging of wild-type and POLG macrophages, the macrophages were cultured in DMEM supplemented with 10% FBS and seeded at a density of 10^5^ cells within 2 ml of the culture media per 35 mm glass-bottom imaging dish 24 h before imaging. Experimental groups included control and cyanide-treated wild-type (WT) and POLG macrophages. Both the WT and POLG macrophages were treated with NaCN (4 mM) to inhibit OXPHOS, and autofluorescence lifetime imaging was performed after 5 min. The NAD(P)H fluorescence lifetime images were captured by a customized built multiphoton imaging system (Mariana, 3i) using a 40× water immersion objective (1.1 NA). The NAD(P)H fluorescence was excited at 750 nm with a power of ∼5 mW using a tunable (680–1080 nm) Ti:sapphire femtosecond laser (COHERENT, Chameleon Ultra II) and detected with a photomultiplier tube (PMT) detector (HAMAMATSU, H7422PA-40) with a bandpass filter (447/60 nm). The fluorescence lifetime decay was measured in the time domain with a time-correlated single-photon counting (TCSPC) electronics module (SPC-150N, Becker & Hickl). Each fluorescence lifetime image (256 × 256 pixels, 270 × 270 *μ*m^2^) was acquired with a pixel dwell time of 50 *μ*s and 5 frame repeats. The NAD(P)H fluorescence lifetime images were analyzed by SPCImage (Becker & Hickl) to calculate the mean NAD(P)H lifetime (*τ_m_*) and export the NAD(P)H intensity image and temporal point spread function (TPSF) image (256 × 256 × 256) with a spatial binning of 9 pixels. The mean lifetime NAD(P)H images (*τ_m_*) were created in SPCImage for visualization of the lifetime, but the CNN models in this paper only use the raw lifetime decay data. The NAD(P)H fluorescence decay image of each cell was obtained by segmentation based on the cell mask generated from NAD(P)H intensity images using Cellpose.^55,56^ The resulting number of macrophages imaged for each group is summarized in Table S2.

### C. Pre-processing and down-sampling of TPSF images

**FIG. 5.**
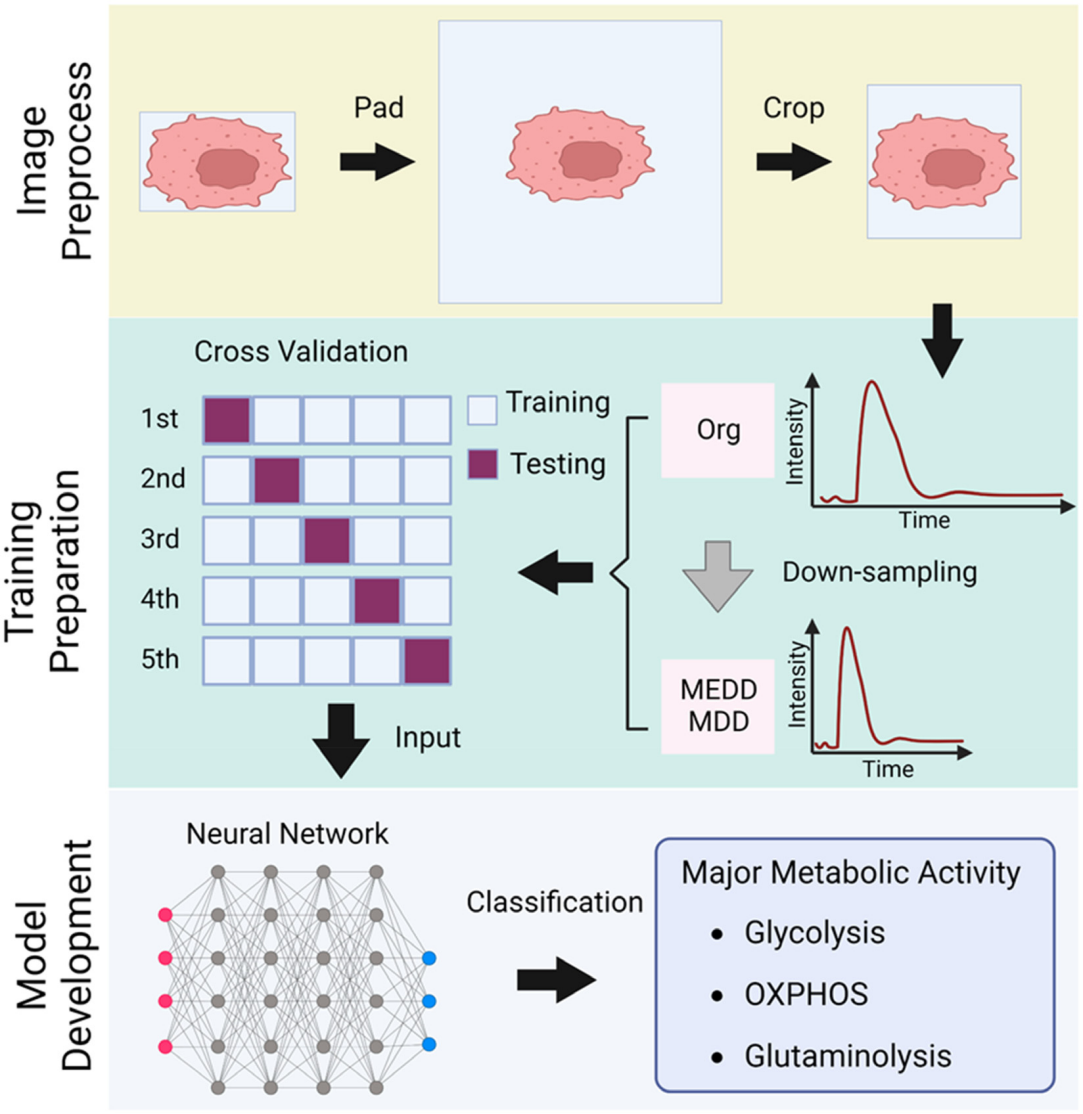
FLIM image pre-processing steps and overview of model development (created with BioRender.com). Each cropped image of unique spatial dimensions × 256 time frames pixels is padded to a uniform 40 × 40 × 256 pixel image and cropped to 21 × 21 × 256 pixels. The cropped images are retained in the original (Org) dimensions (21 × 21 × 256) and down-sampled in the time dimension via either a median (MEDD) or mean (MD) filter to 21 × 21 × 128 pixels. Representative TPSF curves demonstrate the effect of the down-sampling procedure. The resulting dataset of ∼7500 cells is divided into training and test groups for CNN model development to identify cellular metabolic activity from the FLIM images.

Each cell within each NAD(P)H FLIM image was extracted based on the bounding box of its cellular mask to obtain an X-Y-T TPSF image using MATLAB. Then, the following image processing steps were performed in Python with the OpenCV package. The overall workflow for TPSF image processing, training preparation, and CNN model development is described in Fig. 5. First, the pixel values in the cellular regions of each time frame image were summed to plot the photon distribution as a function of time for each TPSF image. Then, the cells were filtered using an entropy threshold at the time frame with the maximum photon number to remove incomplete or poorly segmented cells. The thresholds were defined based on the distribution of entropy using a Gaussian approximation.^34^ To unify the image size, all cell images were padded with borders of 0-values to be 40 × 40 spatial (X-Y) pixels for all 256-time points (T) (Fig. 5). The collective 40 × 40 × 256 TPSF images of ∼7500 cells occupied ∼4 gigabytes of memory. To eliminate the background pixels from the TPSF images and reduce the amount of data, a 21 × 21 × 256 square was extracted in the center of the original image (40 × 40 × 256) to preserve the key morphology of the cells in the spatial domain (Fig. 5). This cropping size (21 × 21 pixels) was selected by analysis of the cell size distribution of the dataset (Fig. S1). Two down-sampling modalities were developed to further reduce each image size to 21 × 21 × 128 pixels while maintaining spatial and temporal information. First, mean and median filters with a window of three pixels in the time domain were applied to the original TPSF images to smooth the decay curve [Fig. S2(a)]. Then, the odd time frames were extracted as the down-sampled data [Fig. S2(a)]. The TPSF images down-sampled by the median filter are defined as MEDD (Median Down-sampled), and those down-sampled by the mean filter are defined as MD (Mean Down-sampled).

### D. CNN model building, training, and evaluation

Two different CNN architectures were developed to predict metabolic phenotypes of cancer cells. All models were created using the machine-learning library Keras with a TensorFlow backend in Python running on Jupyter Notebook on the platform Anaconda. First, a 3D LeNet architecture^36^ with two convolutional layers and two pooling layers was applied to build a model (FLI-LeNet) to predict cells as either glycolytic or oxidative phenotypes. Then, another 3D LeNet model (FLI-LeNet) was trained to perform three metabolic group classification: those dependent on glycolysis, OXPHOS, or glutaminolysis. The second CNN architecture was developed based on the residual neural networks (ResNet) structure,^49^ consisting of a convolutional layer followed by two ResNet blocks. Each ResNet block is comprised of two convolutional layers, and the weight layer learns residual functions by comparing them to the layer inputs. The ResNet CNN model (FLI-ResNet) was created to identify three metabolic activities of cancer cells: glycolysis, OXPHOS, and glutaminolysis. The parameters of the 3D CNN models are described in detail in the supplementary material.

For all models, the size of the input layer was 21 × 21 × 256 for models trained with the original data or 21 × 21 × 128 for models trained with down-sampled data. Cross entropy was set as the loss function and monitored in each training epoch. The Adam optimizer was used with an initial learning rate set to 10^−3^ and a batch size of 8. As a preliminary test, the networks were normally trained for 100 epochs using an NVIDIA GeForce RTX 3080 GPU. 70% of the TPSF images were randomly selected as the training dataset, 10% of TPSF images were used as the validation dataset to monitor the performance of models during training, and the remaining 20% of TPSF images were selected as the testing dataset (Table S1). The training time varied slightly, between 30 and 60 s per epoch, depending on the batch size and original or down-sampled datasets.

### E. CNN model evaluation

Once the best training parameters were determined, a fivefold cross-validation of the models trained to 50 epochs was applied to evaluate the robustness of the CNN models (Fig. 5). The prediction performance of the test datasets was averaged across the fivefold validation. For the prediction of glycolytic cells and oxidative cells, the results were presented in a confusion matrix, where glycolytic cells were defined as the positive group, and the oxidative cells were defined as the negative group. The accuracy was calculated as the ratio of correctly classified cells to the total number of cells. The precision was calculated as the ratio of true positives to the sum of true positives and false positives, and the recall was calculated as the ratio of true positives to the sum of true positives and false negatives. Additionally, a receiver operating characteristic (ROC) curve and the area under the ROC curve (AUC) were obtained from the prediction results of the test datasets for each classifier.

For reporting the performance of the models to distinguish among three metabolic pathways, the precision for each class was calculated as the proportion of correctly predicted cells of that class out of all cells predicted as that class. The recall for each class was calculated as the proportion of correctly predicted instances of that class out of all actual instances of that class in the dataset. The F1-score for each class was calculated by the harmonic mean of precision and recall using the formula F1−score=2 *(Precision * Recall)/(Precision+Recall). An overall precision, recall, and F1-score were obtained by averaging the precision, recall, and F1-score for each class.

After the development and validation of the models on MCF7 cancer cell data, the two-class FLI-LeNet models were applied to the WT and POLG macrophage FLIM images to predict the major metabolic activity for each cell. From the model outputs, the percentage of cells predominantly utilizing glycolysis or OXPHOS was calculated for both the WT and POLG macrophages and their respective cyanide-treated groups, to facilitate a comparison of the metabolic activities among the different groups.

## SUPPLEMENTARY MATERIAL

See the supplementary material for a detailed description of fluorescence lifetime analysis, CNN development, BMDM preparation, Seahorse assay, dataset, and CNN performance.

## Data Availability

The data that support the findings of this study are available from the corresponding author upon reasonable request.
